# Clinical relevance of microhemorrhagic lesions in subacute mild traumatic brain injury

**DOI:** 10.1007/s11682-017-9743-6

**Published:** 2017-06-29

**Authors:** H. J. van der Horn, S. de Haan, J. M. Spikman, J. C. de Groot, J. van der Naalt

**Affiliations:** 1 Department of Neurology, University of Groningen, University Medical Center Groningen, Hanzeplein 1, 9700 RB Groningen, The Netherlands; 2 Department of Neuropsychology, University of Groningen, University Medical Center Groningen, Groningen, The Netherlands; 3 Department of Radiology, University of Groningen, University Medical Center Groningen, Groningen, The Netherlands

**Keywords:** Mild traumatic brain injury, MRI, Microhemorrhages, Microbleeds, Post-traumatic complaints

## Abstract

Magnetic resonance imaging (MRI) is often performed in patients with persistent complaints after mild traumatic brain injury (mTBI). However, the clinical relevance of detected microhemorrhagic lesions is still unclear. In the current study, 54 patients with uncomplicated mTBI and 20 matched healthy controls were included. Post-traumatic complaints were measured at two weeks post-injury. Susceptibility weighted imaging and T2*-gradient echo imaging (at 3 Tesla) were performed at four weeks post-injury. Microhemorrhagic lesions (1–10 mm) were subdivided based on depth (superficial or deep) and anatomical location (frontal, temporoparietal and other regions). Twenty-eight per cent of patients with mTBI had ≥1 lesions compared to 0 % of the healthy controls. Lesions in patients with mTBI were predominantly located within the superficial frontal areas. Number, depth and anatomical location of lesions did not differ between patients with and without post-traumatic complaints. Within the group of patients with complaints, number of complaints was not correlated with number of lesions. In summary, microhemorrhages were found in one out of four patients with uncomplicated mTBI during follow-up at four weeks post-injury, but they were not related to early complaints.

## Introduction

Frequently, no abnormalities are found on computed tomography (CT) in the acute phase after mild traumatic brain injury (mTBI) (G L Iverson et al. 2000). When patients suffer from (persistent) cognitive complaints interfering with daily activities, magnetic resonance imaging (MRI) is routinely performed to assess traumatic parenchymal abnormalities. However, studies have shown that the number of self-reported symptoms (which we will refer to as post-traumatic complaints) in the sub-acute phase after mTBI does not differ between patients with and without lesions on admission CT and/or follow-up MRI (i.e. complicated vs. uncomplicated mTBI) (Grant L. Iverson et al. 2012; Panenka et al. 2015). Microhemorrhagic lesions are among the most frequently found traumatic abnormalities in mTBI, especially due to the sensitivity of susceptibility weighted imaging (SWI) and to a lesser extent of T2*-gradient echo (GRE) imaging (Huang et al. 2015; Yuh et al. 2013). However, the clinical relevance of these microhemorrhages in addition to other traumatic lesions, with regard to post-traumatic complaints (Hofman et al. 2001; Hughes et al. 2004), cognitive performance (Hofman et al. 2001; Huang et al. 2015; Hughes et al. 2004; Lee et al. 2008) and outcome (Yuh et al. 2013), is still unclear. Hence, clear criteria to make a distinction between clinically relevant and non-relevant lesions in mTBI are currently not available. This pertains not only to the number of lesions, but also to depth and anatomical location of these lesions.

The aim of the current study was to gain more insight into the number, depth and anatomical location of microhemorrhages on SWI and T2*-GRE in patients with uncomplicated mTBI and healthy controls, and to find clues for the interpretation in clinical practice, especially with regard to the presence or absence of post-traumatic complaints at two weeks post-injury.

## Methods

### Participants

This study is part of a larger prospective multicentre cohort study on outcome post-mTBI (UPFRONT study) conducted between March 2013 and February 2015. Patients were included at the emergency department (ER) of the University Medical Centre Groningen (a level I trauma centre). Inclusion criteria for the MRI study were: age between 18 and 65, no abnormalities on admission CT-scan (i.e. uncomplicated mTBI), and the presence or absence of post-traumatic complaints at two weeks post-injury. Exclusion criteria were: major neurologic or psychiatric co-morbidity, admission for prior TBI, drug or alcohol abuse, mental retardation. This information was obtained from the patients’ history at the ER or neurology ward, and through questionnaires at two weeks post-injury. Contraindications for MRI were: implanted ferromagnetic devices or objects, pregnancy or claustrophobia. Twenty age, sex and education matched healthy controls without a history of TBI were recruited among social contacts and via advertisements.

The study was approved by the Medical Ethics Committee of the UMCG; all participants provided informed consent.

### Clinical measures

A head injury complaints checklist, consisting of 19 post-traumatic complaints, was administered at two weeks post-injury to patients, but not to healthy controls (de Koning et al. 2016). This is a sensitive questionnaire, which also corrects for pre-injury complaints. Having complaints was defined as ≥3 complaints with at least one complaint in the cognitive and/or affective domain (de Koning et al. 2016; Dischinger et al. 2009; Lundin et al. 2006; Matuseviciene et al. 2015; McMahon et al. 2014).

### MRI acquisition

Approximately four weeks post-injury 3 T MRI scans (Philips Intera with a 32 channel SENSE head coil, Philips Medical Systems, Best, the Netherlands) were made comprising the following sequences: transversal T1 (TR 9 ms; TE 3.5 ms; FA 8°; FOV 256 × 232 mm; voxel size 1x1x1 mm), coronal T2*- GRE (TR 875 ms; TE16ms; FOV 230 × 183.28 mm; voxel size 0.40 × 1.12x4mm) and transversal SWI (venous BOLD: TR 35 ms; TE 10 ms; FOV 230 × 183.28 mm; voxel size 0.90 × 0.90x2mm).

### Scoring

Classification of microhemorrhagic lesions was based on previously published guidelines for scoring primary (non-traumatic) microbleeds: 1) black round or oval lesions with blooming effect on T2*-GRE, 2) devoid of signal hyperintensity on T1- or T2-weighted sequences, 3) at least half surrounded by brain parenchyma, 4) distinct from mimics such as iron/calcium deposits, bone, or vessel flow voids (Greenberg et al. 2009), similar to the study by Huang and colleagues (Huang et al. 2015). Hypointensities that were difficult to classify were scored as indeterminate. Lesions (1–10 mm in diameter) were scored according to number, depth (superficial (cortical and juxtacortical) vs. deep (subcortical), adjusted from (Huang et al. 2015)) and anatomical location (frontal, temporal, parietal, occipital, insula/basal ganglia, thalamus, corpus callosum). Infratentorial lesions were classified into a separate category. For analyses, anatomical locations were trichotomized into: frontal, temporoparietal and other. Images were scored independently by a senior medical student (S.d.H.) and a neuroradiologist (J.C.d.G.), who were blinded for group label. Presence of lesions was determined primarily using transversal SWI. In addition, coronal T2*-GRE was used to examine regions in proximity of the skull base. In 73% of cases there was initial concordance between the researchers. Disconcordance mostly concerned small (1–2 mm) superficial hypointensities, scored as microhemorrhages or as vessel flow voids. These cases were reanalyzed for definitive concordance.

Lesion characteristics were compared between healthy controls and patients with mTBI, and between patients with and without complaints. Correlations were computed between number of lesions and number of complaints.

### Statistics

Data were analysed using IBM Statistical Package for the Social Sciences (SPSS) version 22. Normality was assessed using Shapiro-Wilk tests. Because all continuous variables (age, lesion numbers and complaint scores) followed a non-parametric distribution, Mann-Whitney *U* tests were used for group comparisons. Chi square tests were used for nominal (sex, frequencies of patients with positive MRI) and ordinal (education level) variables. Spearman’s rank correlations were used for correlation analyses between number of lesions and number of complaints. Significance was set at α = 0.05. Bonferroni corrections were used for multiple comparisons.

## Results

### Participant characteristics and clinical measures

Participant characteristics are listed in Table 1. The group of patients without complaints (*n* = 20) contained more male participants compared to patients with complaints (*n* = 34; *p* = 0.005). No further differences were found between patient subgroups. On average, patients with complaints reported 10 complaints. Fatigue (88%), headache (85%) and noise intolerance (85%) formed the top 3 most frequently reported complaints for the PTC-present group.

**Table 1:** Participant characteristics

	mTBI (*n* = 54)	HC (*n* = 20)	*p*-value
Age, mean (range), years	37 (19–64)	34 (18–61)	0.559
Sex, % male	67	65	0.893
Education level, median (range)^a^	6 (2–7)	6 (5–7)	0.110
Interval injury to MRI, median (range), days	33 (22–69)	N/A	N/A
GCS-score, median (range)	15 (13–15)	N/A	N/A
Injury mechanism:			
Traffic, % of group	50	N/A	N/A
Falls, %	42	N/A	N/A
Sports, %	2	N/A	N/A
Assault, %	2	N/A	N/A
Other, %	4	N/A	N/A

^a^Education level was based on a Dutch classification system, according to Verhage (Verhage 1964), ranging from 1 to 7 (highest).

*HC* healthy controls, *MRI* Magnetic Resonance Imaging, *mTBI* mild traumatic brain injury, *N/A* not applicable, *GCS* Glasgow Coma Score.

### Lesions in mTBI and healthy controls

Total number of lesions in the mTBI group was 158 compared to zero lesions in healthy controls. In 28% (*n* = 15) of patients with mTBI at least one lesion was present: one lesion in 7%, two lesions in 4%, three lesions in 2%, and ≥4 lesions in 15%. Of the total number of lesions, 71% were located in the superficial areas. Regardless of depth, 70% of lesions were located within the frontal lobes, 15% in the temporoparietal lobes and 15% in other areas. Two patients had three corpus callosum lesions (both had in total ≥ 4 lesions). Regarding the group of patients with ≥1 lesions, 40% (*n* = 6) of the group had lesions merely located in the frontal areas (sum = 30 lesions), 40% (*n* = 6) had lesions in both frontal as well as in areas outside the frontal lobes (sum = 125 lesions; 80 in frontal, 20 in temporoparietal and 25 in other regions), and 20% (*n* = 3) had lesions that were located merely outside the frontal lobes (sum = 3).

In 17% of patients with mTBI (total of 23 lesions) and 10% of healthy controls (total of 2 lesions, 1 located in the temporal and 1 in the infratentorial region) indeterminate lesions were scored. In patients with mTBI, 48% of indeterminate lesions were located in the superficial frontal areas, 35% in the superficial parietal areas, and 17% in other areas.

### Lesions and post-traumatic complaints

**Fig. 1:**
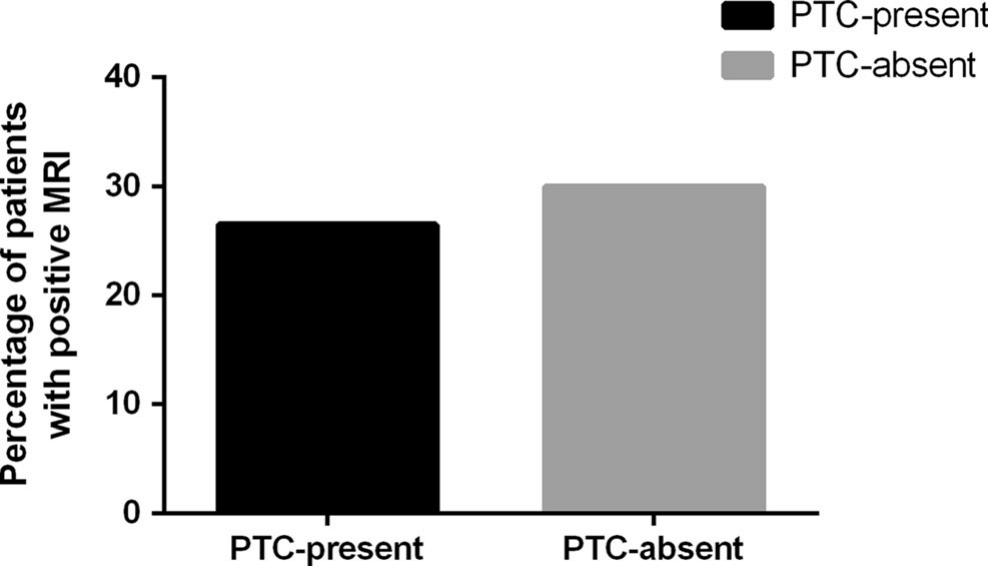
Percentage of patients with ≥1 microhemorrhages

**Fig. 2:**
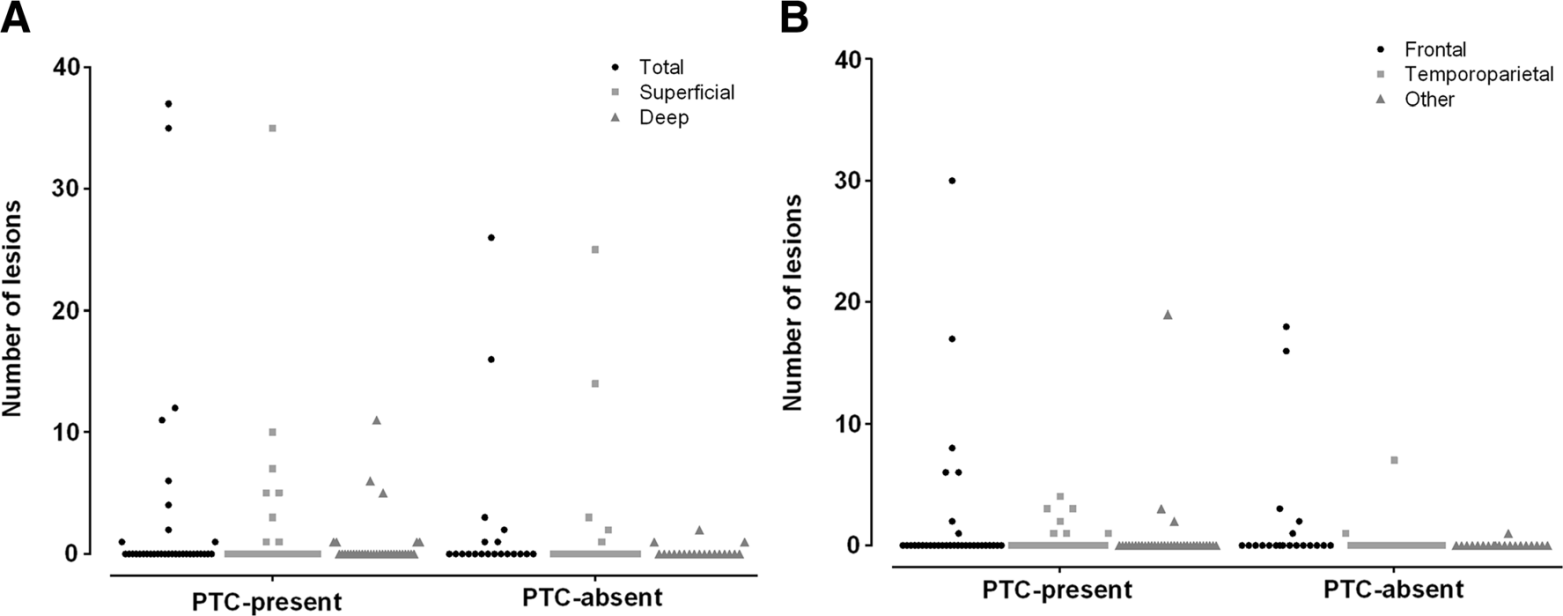
Number of lesions related to depth (**a**) and anatomical location (**b**) in (individual) patients with (PTC-present) and without (PTC-absent) post-traumatic complaints at two weeks post-injury

The percentage of patients with one or more microhemorrhages on MRI was not significantly different between patients with and without complaints (26.5 versus 30%; χ^2^ = 0.078; *p* = 0.78; Fig. 1). No significant differences in number of lesions were found between patients with and without complaints (109 versus 49; *U* = 334, *p* = 0.883) (Fig. 2a). No group differences in number of superficial (*U* = 336, *p* = 0.914), deep (*U* = 319, *p* = 0.569) and indeterminate (*U* = 322, *p* = 0.619) lesions were present. Regarding anatomical location, no group differences were present for number of lesions in frontal (*U* = 328, *p* = 0.758), temporoparietal (*U* = 306, *p* = 0.341) or other regions (*U* = 326, *p* = 0.581) (Fig. 2b). Furthermore, the percentages of patients with ≥1 lesions in frontal (χ^2^ = 0.14, *p* = 0.71), temporoparietal (χ^2^ = 1.02, *p* = 0.313), or other (χ^2^ = 0.27, *p* = 0.6) regions were evenly distributed over the groups with and without complaints. This was also true for the percentages of patients with ≥1 lesions merely in frontal, and/or in regions outside the frontal lobes (χ^2^ = 3.19, *p* = 0.202).

For patients with complaints, number of lesions was not related to number of complaints (rho = −0.09, *p* = 0.616).

## Discussion

In the present study, clinical relevance of microhemorrhages was investigated with SWI and T2*-GRE imaging in a relatively small sample of patients with uncomplicated mTBI in the subacute phase after injury. One in four patients with mTBI had lesions, mainly located within the superficial frontal areas. Number, depth and anatomical location of lesions were not different between patients with and without complaints at two weeks post-injury. No lesions were found in healthy controls.

Patients and doctors may attribute complaints and problems in the resumption of daily activities to abnormalities found on SWI and T2*-GRE, which are sensitive to detect microhemorrhagic lesions. However, the clinical implication of these lesions is still unclear. It has been shown that the presence of three or fewer hemorrhagic axonal lesions on early T2*-GRE was not related to poorer outcome at three months post-injury, although SWI was not performed (Yuh et al. 2013). A recent study that included a large sample of uncomplicated mTBI patients in the subacute phase post-injury and healthy controls, reported that patients with microhemorrhagic lesions on SWI had lower scores on a working memory task, but not on an attention task, than patients without lesions (Huang et al. 2015). However, test scores in healthy controls were not mentioned, and we know from the literature that most of the patients with mTBI do not have any impairments on neuropsychological tests, while they may still report significant cognitive complaints (Carroll et al. 2014; Rohling et al. 2011). Regarding these post-traumatic complaints, studies so far have not shown differences in number of complaints between mTBI patients with and without lesions on T2*-GRE and SWI in the acute and sub-acute phase post-injury (Hofman et al. 2001; Hughes et al. 2004; Iverson et al. 2012). However, to our best knowledge, the relationship of depth and anatomical location of microhemorrhages to complaints after mTBI has not been examined with neither T2*-GRE nor with SWI.

In the current study, the majority of patients with mTBI (72%) had normal SWI and T2*-GRE scans at four weeks post-injury, which is consistent with others (Huang et al. 2015; Yuh et al. 2013), although our study sample was not entirely representative of the general mTBI population. Number of lesions (superficial and deep) was similar for patients with and without complaints. Most of the lesions were located within the frontal lobes, which is in line with previous diffusion tensor imaging studies (Eierud et al. 2014); but again, lesions in these regions were not associated with the presence of post-traumatic complaints. These findings might indicate that the underlying micro-structural pathology of mTBI and post-traumatic complaints is not, or not accurately, reflected by the presence of microhemorrhages.

Small superficial microhemorrhagic lesions are easily detected with MRI due to the high sensitivity of especially susceptibility weighted sequences, although lesions are difficult to discriminate from flow void artefacts (Greenberg et al. 2009). Here, one fifth of patients with mTBI had indeterminate lesions, mainly located in the superficial cerebral areas. Since these small (1–2 mm) lesions were found to have no relation with post-traumatic complaints, this may suggest that MRI results in overrating of (irrelevant) lesions in clinical practice.

To conclude, in one out of four patients with mTBI microhemorrhagic lesions were present on MRI. Most interestingly, the presence, number, depth and localization of lesions were not related to the presence and number of complaints after mTBI, which may question whether MRI with SWI and T2*-GRE sequences should be made routinely in clinical practice in patients with persistent complaints. We realize that the relatively small sample size limits overall generalizability of results. Further work is required to extend our findings, and to determine the exact relationships of microhemorrhages to underlying neural injury.
